# Production of Antioxidants and High Value Biomass from *Nannochloropsis* *oculata*: Effects of pH, Temperature and Light Period in Batch Photobioreactors

**DOI:** 10.3390/md20090552

**Published:** 2022-08-29

**Authors:** Vasilis Andriopoulos, Fotini N. Lamari, Sophia Hatziantoniou, Michael Kornaros

**Affiliations:** 1 Laboratory of Biochemical Engineering & Environmental Technology (LBEET), Department of Chemical Engineering, University of Patras, 26504 Patras, Greece; 2 Department of Pharmacy, University of Patras, 26504 Patras, Greece

**Keywords:** antioxidant, biorefinery, carbohydrate, DHA, EPA, lipid, pigment

## Abstract

*Nannochloropsis oculata* is a marine microalgal species with a great potential as food or feed due to its high pigment, protein and eicosapentaenoic acid contents. However, for such an application to be realized on a large scale, a biorefinery approach is necessary due to the high cost of microalgal biomass production. For example, techno economic analyses have suggested the co-production of food or feed with antioxidants, which can be extracted and supplied separately to the market. The aim of this study was to investigate the effect of cultivation conditions on the antioxidant capacity of *Nannochlosopsis oculata* extracts, derived with ultrasound-assisted extraction at room temperature, as well as the proximate composition and fatty acid profile of the biomass. A fractional factorial approach was applied to examine the effects of temperature (20–35 °C), pH (6.5–9.5) and light period (24:0, 12:12). At the end of each run, biomass was collected, washed with 0.5M ammonium bicarbonate and freeze-dried. Antioxidant capacity as gallic acid equivalents as well as pigment content were measured in the ethanolic extracts. Optimal conditions were different for productivity and biomass composition. Interesting results regarding the effect of light period (LP) and pH require further investigation, whereas the effect of moisture on the extraction process was confounded with biomass composition. Finally, further data is provided regarding the relation between chlorophyll content and apparent phenolic content using the Folin–Ciocalteu assay, in agreement with our previous work.

## 1. Introduction

*Nannochloropsis* is a genus of the class Eustigmatophyceae, comprised of four marine and one freshwater species. Two former marine members of *Nannochloropsis* now comprise the new genus *Microchloropsis* [1]. Among the marine species of *Nannochloropsis*, *Nannochloropsis oculata* has been proposed to produce Eicosapentaenoic Acid (EPA) and used as aquaculture feed. Protein content per dry weight of *N. oculata* ranges between 25% and 60%, carbohydrate content between 1% and 20% and lipid content between 8% and 50%. EPA content in terms of dry weight seems to be relatively independent from total lipid biomass fraction [1] and ranges between 3% and 6 % of dry weight [2]. Regarding the pigments, the genus *Nannochloropsis* contains only chlorophyll a (Chla), with a distinctive absence of chlorophyll b (Chlb) and chlorophyll c (Chlc), whereas the prevalent carotenoids are xanthophylls [3]. *Microchloropsis* species can accumulate astaxanthin as their primary carotenoid [2], whereas *N. oculata* contains primarily violaxanthin [3]. 

Various polar and non-polar solvents have been used to extract pigments and antioxidant compounds such as phenolic and flavonoids from microalgae, with methanol being the most common. Total phenolic content measured with the Folin–Ciocalteu assay and expressed in terms of dry weight for *Nannochloropsis* is 2.9–8 mg Gallic Acid Equivalents per g Dry Weight (GAE g^−1^ DW) [4], with acetone being the most efficient solvent, followed by methanol and water being the worst. Antioxidant power in terms of 2,2-diphenyl-1-picrylhydrazyl (DPPH) or (2,2′-azino-bis(3-ethylbenzothiazoline-6-sulfonic acid)) (ABTS) scavenging activity for *Nannochloropsis* ranges from 0 to 6.5 mg TE g^−1^ DW [5], and reduces potential in terms of ferric reducing power from 0.3 to 10.2 mg TE g^−1^ DW [5]. The types of molecules that can contribute to these activities include pigments, phenolics, flavonoids, fatty acids and vitamins. *Nannochloropsis oculata* extracts have also demonstrated anti-inflammatory, antidiabetic and apoptotic activities [6,7,8,9,10], whereas over 400 proteins have been identified as potential candidates against cancer in *Nannochloropsis gaditana* using proteomics [11].

Most optimization studies for *Nannochloropsis oculata* have focused on biodiesel production [12,13,14] or the effects of nutrient ratios in the medium [15,16,17]. In this study, we decided to investigate the effects of temperature, pH and light period on the biomass composition, fatty acid profile and quality of ethanolic extracts, as a continuation of previous work for the production of antioxidants and aquaculture feed from marine microalgae [3,4]. Temperature affects cell growth and composition either directly by affecting protein stability and function and membrane fluidity or indirectly by controlling gas mass transfer rates of CO_2_, O_2_ and ammonia. However, *N. oculata* can grow in a wide range of temperatures from 5 to 35 °C with an optimal for growth at ~20 °C [18] and optimal for lipid production at 30 °C [19]. Unsaturated fatty acids such as EPA, however, accumulate at low temperatures [20]. pH affects the availability of CO_2_ via the bicarbonate equilibrium whereas at high pH ammonium it dissociates to free ammonia, leading to both loss of nitrogen from the medium and, in some cases, inhibition of photosynthesis. *N. oculata* however seems to be unaffected by increased ammonia concentrations [21]. High pH values can also cause stripping of phosphates via precipitation [22]. When CO_2_ is a limiting factor, the utilization of bicarbonate via carbon concentrating mechanisms can increase pH [23]. Uptake of nitrate and ammonium increase and decrease pH, respectively [24]. *N. oculata* can grow in a range of pH between 6.5 and 9.5 with an optimal for growth at ~8 [18]. Salinity is an important abiotic parameter for microalgae cultivation that has been less studied than temperature, pH and light intensity. Both hyposalinity and hyper-salinity have an effect of cell growth rate, cell volume and biomass composition, especially on lipid fraction and composition. In *Nannochloropsis*, the lipid fraction generally increases with decreasing salinity, with the same applying to the EPA lipid fraction [25]. Salt stress has also been used to increase carotenoid content, especially in freshwater species such as *Haematococcus pluvialis* [26]. In *N. oculata,* the effect of increasing NaCl concentration to carotenoid content is similar to that of copper stress [27], which is consistent with generation of ROS during increased ion concentration in the cell. Light availability in autotrophic microalgae cultures is the factor which puts a limit on biomass density that can be achieved. Under low light, cells increase their pigment content making light penetration even more difficult. Below a certain threshold, photosynthesis can no longer sustain cell growth and biomass loss is observed. On the other hand, at high light intensity, the photosynthetic yield on photons decreases and above a certain threshold increasing light intensity causes photooxidation and results in bleaching and death of the cells. Under high light conditions, microalgae respond by either reducing the antenna size or the number of photosynthetic units, with the literature indicating that *Nannochloropsis* follows the second strategy [28]. Additionally, *Nannochloropsis* responds to high light by increasing carotenoid content, which could directly scavenge the ROS generated [29]. Light period, meaning the ratio of time a culture is exposed to light and dark, plays a significant role, especially under saturating light intensities when dark periods can improve biomass growth by limiting photoinhibition. In *Nannochloropsis,* moderate light intensity and photoperiod 16:8 increased biomass concentration and lipid content [30].

The aim of this study was the investigation of the effects of light period, pH and temperature on the growth and biochemical composition of *N. oculata,* especially in terms of pigments, proteins, and EPA content, as a continuation of our previous work on the development of a process for the co-production of aquaculture feed and antioxidant extracts from marine microalgae [3,4].

## 2. Results

### 2.1. Growth

**Figure 1:**
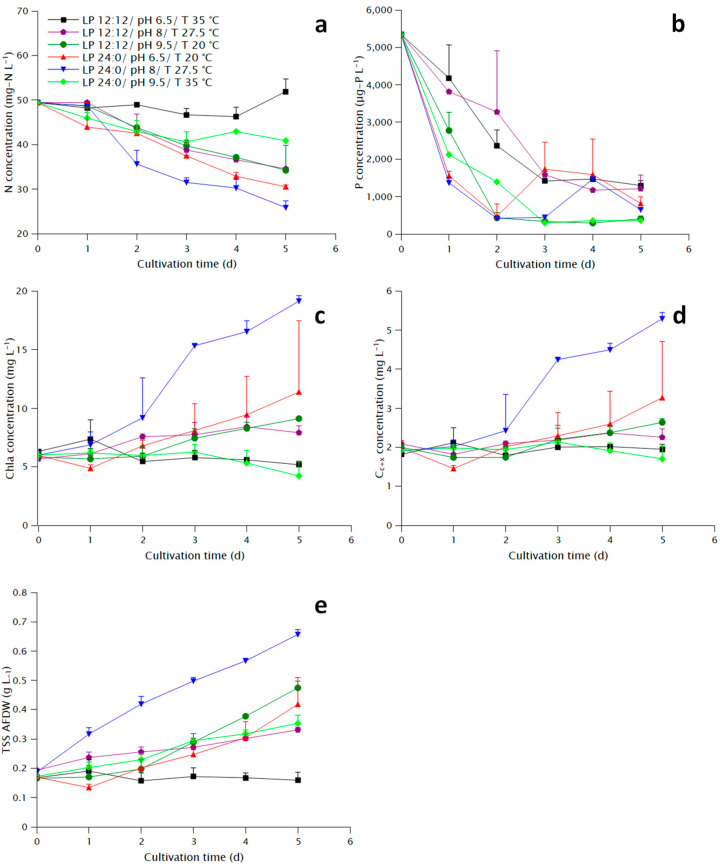
Growth parameters during the cultivation of *N. oculata* under different conditions: (**a**) Nitrate concentration, (**b**) Total phosphate concentration, (**c**) Chla concentration, (**d**) Carotenoid and xanthophyll (Cc+x) concentration, (**e**) Biomass concentration measured as ash-free total suspended solids (TSS AFDW).

The highest maximum specific growth rate, final biomass density and pigment concentration was displayed by condition E (Table 1). The lowest maximum specific growth rate and final biomass density were presented by condition A, whereas the lowest pigment concentration by condition F. Condition D displayed a lag phase as can be seen both in the TSS and the pigment plots (Figure 1c–e). Growth in terms of TSS in conditions C and F (pH 9.5) was not delayed, however, the precipitation due to the addition of NaOH might have caused an overestimation of the biomass concentration, although the ash-free dry weight (AFDW) was used to resolve that issue. A better indicator for those conditions would be the pigment concentration which for condition F showed a declining pattern, the same as condition A (also 35 °C), whereas condition C showed a similarity to condition B. 

The measurement of nitrate during the last two days displayed large standard deviation (Figure 1a), probably due to interference of organic compounds released from the cells with the colorimetric method described in Section 4.3, and thus no significant differences were found between different conditions apart from condition A, where it was practically zero, and the rest. However, the evolution of nitrate concentration clearly shows more rapid consumption for conditions D and E compared to the rest. Similarly, conditions D and E showed the most rapid phosphate consumption (Figure 1b). The apparent consumption of phosphate in conditions C and F might be more related to precipitation due to the addition of NaOH than uptake from the cells. The increase in phosphate concentration at day 3 and 4 for conditions D and F, respectively, might be related to release of phosphate-containing organic compounds from the cells, which would also cause the interference to the nitrate concentration measurement. In general, nitrate consumption reflected the observed growth, whereas phosphate uptake seems to be correlated to the light period, with a much steeper curve at the conditions with continuous light.

### 2.2. Biomass Composition

Condition B contained significantly more carbohydrates than the rest of the conditions, as well as the highest protein, EPA and Chla contents along with condition D, and the lowest fraction of unidentified (Other) compounds (Table 2). Condition Ε presented the highest C_c+x_ content with conditions A and B following. The fatty acid content present at condition C was significantly lower than that at all other conditions except for condition F (also pH 9.5). In general, pH 9.5 negatively affected carbohydrate, protein, lipid and pigment content while increasing the fraction of unidentified compounds and photoperiod 12:12 had a positive influence on carbohydrate and EPA content.

### 2.3. Fatty Acid Profile

**Figure 2:**
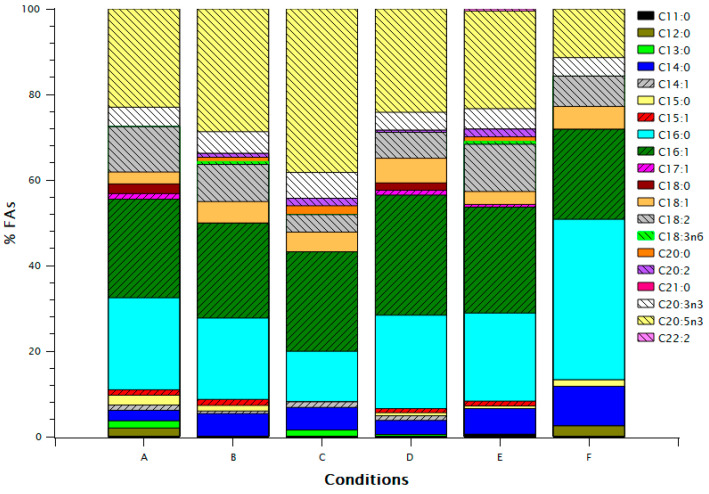
FA (Fatty Acid) profile for the different growth conditions. Concentrations of FAs are expressed as percentage of total FAs.

Condition C presented the highest EPA and lowest C16:0 fractions, opposite to condition F (Figure 2), despite the two conditions having approximately the same FA biomass content (Table 2). Conditions D and E had similar FA profile with major differences in C18:1 and C18:2. Conditions A and B had a similar profile to D and E, respectively, with a higher EPA fraction in B than E.

### 2.4. Extraction

The highest and lowest Chla, C_c+x_ and GAE contents in extracts were presented by conditions B and F, respectively (Table 3). Chla and and C_c+x_ extraction yields were highest for condition C and lowest for condition E. In general, there was significant variance in the extraction, reflected by the high standard deviation in some cases, indicating effects of parameters other than growth conditions.

### 2.5. Linear Regression Results

Growth parameters, biomass composition and extraction parameters were analyzed with linear regression in Minitab. Additionally, linear regression was used to identify factors influencing the extraction process. Results are presented below.

#### 2.5.1. Effects of Growth Conditions on Growth Parameters

**Figure 3:**
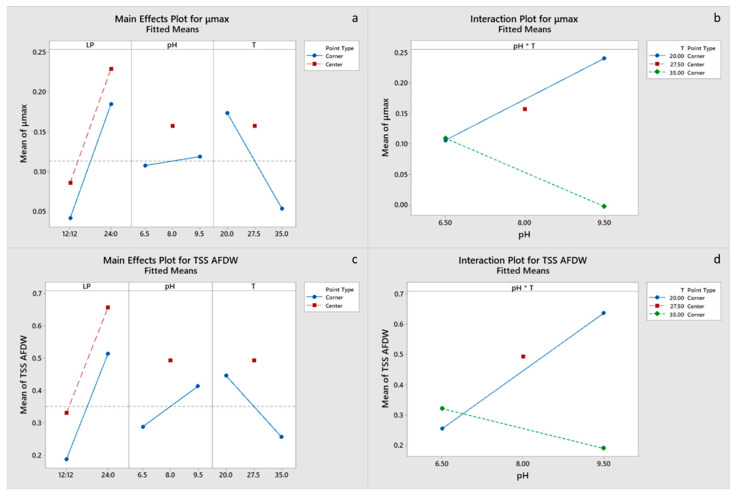
(**a**,**b**): Factorial plots of main effects and interactions, respectively, for μ_max_. (**c**,**d**): Factorial plots of main effects and interactions, respectively, for TSS AFDW.

Specific maximum growth rate was positively influenced by light period and negatively by temperature (Figure 3a,b). Center point did not have a significant effect in the model, indicating a linear relationship between response and variables, and thus the highest growth rate would be expected at low temperatures and continuous light. The model for final N consumption was not significant. Continuous light had a positive effect on final P consumption, although only pH effect was significant, with a higher value for pH 9.5, which is probably related to precipitation. Significant effects on final biomass concentration were the positive effect of continuous light, negative effect of temperature and in a lesser degree the effects of pH-T interaction and pH (Figure 3c,d). Cla and C_c+x_ concentrations were primarily influenced by the light period and pH-T interaction (Supplementary material, Figures S1 and S2). Final biomass and Chla and C_c+x_ concentrations had significant interaction of the center point terms (Supplementary material, Tables S4–S6). However, the estimated optimal conditions for growth are LP 24:0, pH 9.5 and a temperature of 20 °C (Table 4). Factorial plots of the effects included in the models for μ_max_, TSS and pigment concentrations are shown in Figure 3, whereas the regression models are provided in the supplementary materials. 

#### 2.5.2. Effects of Growth Conditions on Biomass Composition

**Figure 4:**
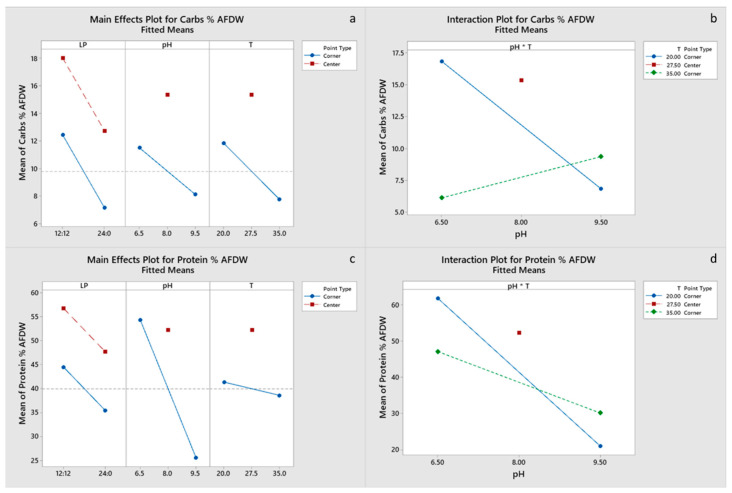
(**a**,**b**) Factorial plots of main effects and interactions, respectively, for carbohydrate biomass content. (**c**,**d**) Factorial plots of main effects and interactions, respectively, for protein biomass content.

**Figure 5:**
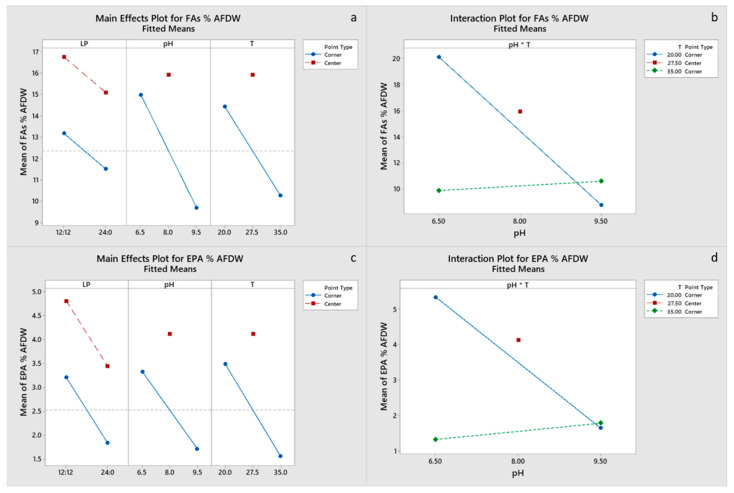
(**a**,**b**) Factorial plots of main effects and interactions, respectively, for FA biomass content. (**c**,**d**) Factorial plots of main effects and interactions, respectively, for EPA biomass content.

**Figure 6:**
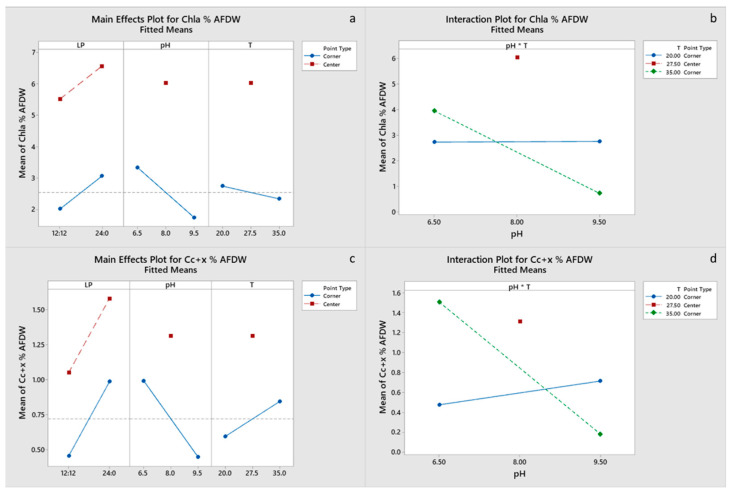
(**a**,**b**) Factorial plots of main effects and interactions, respectively, for Chla biomass content. (**c**,**d**) Factorial plots of main effects and interactions, respectively, for C_c+x_ biomass content.

All main effects and the pH-T interaction were significant for carbohydrate and EPA content of the final biomass, with a negative effect of the light period. Only pH had a significant effect on protein content (Figure 4c,d), whereas pH and the pH-T interaction were significant for lipid content (Figure 5a,b). Chla content was primarily influenced by pH and to a lesser degree by the pH-T interaction, whereas for C_c+x_ light, the period was also significant (Figure 6a–d). Only pH had significant effect on the FA content. Center points had a significant effect on all the biomass components, which suggests that a response surface is more suitable for optimizing the biomass composition. However, the results presented imply that temperature between 20 and 27.5 °C and pH 6.5–8.0 are optimal for protein, pigment and EPA content, whereas the significant positive effect of LP 12:12 on EPA content dictates the need for experimentation with LP in the range 12:12–24:0. Optimal growth conditions for carbohydrate, protein, FA and EPA contents were estimated to be LP 12:12, pH 6.5 and a temperature of 20 °C, for Chl, a content of LP 24:0, pH 8 and a temperature of 27.5 °C, and for C_c+x_ content, LP 24:0, pH 6.5 and a temperature of 35 °C. Factorial plots of the effects included in the models for carbohydrate, protein, FA, EPA, Chla and C_c+x_ biomass contents are shown in Figure 4, Figure 5 and Figure 6, whereas the regression models are provided in the supplementary materials.

#### 2.5.3. Effects on Extraction 

**Figure 7:**
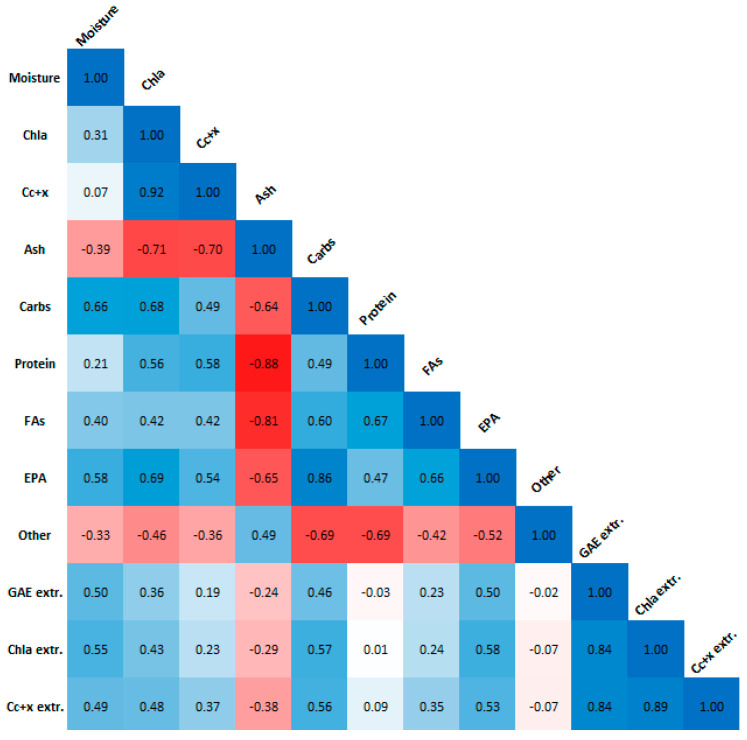
Pearson correlation plot for biomass moisture content and composition, as well as extract Chla, C_c+x_ and GAE content. Absolute values larger than 0.5 indicate strong correlation.

Figure 7 displays the Pearson correlation coefficients for the biomass moisture content and composition, and the extract pigment and GAE contents. A strong positive correlation was found between Chla and C_c+x_ biomass contents, carbohydrate, and EPA biomass contents, and between the extract pigment and GAE contents. A strong negative correlation was found between ash biomass content and Chla, C_c+x_, protein and FA biomass contents. The strong correlation between GAE and Chla agrees with our previous results [4] in that there is a significant interference of chlorophyll on the Folin–Ciocalteu assay, which should not be used on its own for phenolic content estimation in microalgae. Moisture content of the biomass after freeze-drying is correlated to the biomass composition, and thus any effects it has on the extraction process are confounded and need further experimentation to be evaluated. 

## 3. Discussion

Spolaore et al., predicted optimum μ_max_ at 21 °C, low light intensity and pH 8.4 [18]. Converti et al., observed a decrease in growth rate and increase in lipid content with a temperature increase from 20 to 25 °C [12]. Malakootian et al., reported increase of lipid content at 30 °C with uncontrolled pH [19]. Our data support the decrease of growth rate with temperature and a maximum at moderate pH, whereas for lipid content, we found a significant interaction between pH and temperature, with lipid content decreasing at increasing temperatures at low pH, and increasing with increasing temperatures at high pH, and an optimum at low pH and temperature. Since Converti et al., and Malakootian et al., both used nitrate as the nitrogen source, the pH would be alkaline in their cultures, and thus the effect they observed agrees with our results. At pH 6.5, much of the available carbon is in the form of dissolved CO_2_, and thus increase in temperature could lead to decreased gas solubility and CO_2_ availability, and in turn to low carbon availability for production of lipids. In fact, under CO_2_ supplementation, low pH and high temperature might have a positive effect on lipid content [31]. No significant effect of LP was observed for FA content; EPA content, however, was significantly promoted by LP 12:12, confirming the results of Shene et al. [32]. The EPA FA fraction of condition C (38.26%) was very similar to the maximum EPA FA fraction reported by Rasdi and Qin [33] (38.67%) achieved with an N/P ratio 20:1 (molar basis), the same with the current study, light period 12:12 and a temperature of ~21.5 °C. The N/P ratio in the current study was also 20:1. Those authors showed the great influence of the N/P ratio to the FA profile of *N. oculata*, whereas we show the same for the growth condition.

Our observation that carbohydrate and EPA content are highly correlated has not been reported before to our knowledge and requires further investigation. It is known that *Nannochloropsis* accumulates lipids in favor of carbohydrates under nitrogen deprivation [34]; however, the relationship between carbohydrate and fatty acid synthesis during nutrient replete conditions is not very well-studied. Shene et al., observed a decrease in carbohydrate content when shifting the pH from 6.5 to 8 at temperature 20 °C and LP 12:12, and a maximum of Chla and C_c+x_ content at pH 7 [35]. Our results imply that carbohydrate content decreases with a further increase of pH and confirms that Chla displays an optimum close to neutral pH. For C_c+x_, however, the optimal was found at pH 6.5 and a temperature of 35 °C. Kumar and Saramma present similar results for pigments for *N. salina*, with optimal for Chla at pH 9 and for C_c+x_ at pH 6 [36]. In the current study, the effect of high pH was confounded with salinity due to the addition of NaOH. Gu et al., reported a maximum FA biomass content at salinity 25 ppt, which decreased with a further increase of salinity [37]. Gu et al., also reported a decrease of protein and Chla content with salinity, which is in agreement with our results. Finally, the effects of pigments, especially chlorophyll, on the apparent phenolic content of microalgae has been demonstrated in support of our previous work, which also relates DPPH radical scavenging activity and FRAP with GAE and pigment content [4]. Thus, maximization of pigment content in extracts should be the focus of further studies. Apart from the obvious goal of increasing pigment content in the biomass, the possible effect of moisture should also be evaluated. This was not able in this study due to the effect of biomass composition, especially carbohydrates, on the moisture content. Chen et al., reported an optimal moisture content of 10% for lipid extraction from *Nannochloropsis* sp. using subcritical ethanol [38]. In our study, the moisture content varied between 0.76–11.34%.

## 4. Materials and Methods

### 4.1. Chemicals and Reagents 

Macronutrients used were NaNO_3_ (PanReac, Barcelona, Spain) and NaH_2_PO_4_.2H_2_O (Honeywell International Inc., Charlotte, NC, USA), whereas the trace elements used for media preparation were Na_2_EDTA (Sigma-Aldrich, St. Louis, MO, USA), FeCl_3_.6H_2_O (Acros Organics, Geel, Belgium), CuSO_4_.5H_2_O (Sigma-Aldrich, St. Louis, MO, USA), ZnSO_4_.7H_2_O (Sigma-Aldrich, St. Louis, MO, USA), CoCl_2_.6H_2_O (Thermo Fisher Scientific, Pittsburg, PA, USA), MnCl_2_.4H_2_O (Acros Organics, Geel, Belgium) and Na_2_MoO_4_.2H_2_O (Chem-Lab NV, Zedelgem, Belgium). Cyanocobalamin, Thiamine HCl and Biotin were purchased from Sigma-Aldrich, St. Louis, MO, USA.

Chemicals used for analysis were ammonium bicarbonate (Sigma-Aldrich, St. Louis, MO, USA), Folin–Ciocalteu reagent (Sigma-Aldrich, St. Louis, MO, USA), *N*,*N*′-dimethylformamide (DMF) (Honeywell International Inc., Charlotte, NC, USA), chlorophorm HPLC grade (Honeywell International Inc., Charlotte, NC, USA), mercury (III) oxide red (Sigma-Aldrich, St. Louis, MO, USA), 2,4,6-tri(2–pyridyl)–s–triazine (TPTZ) (Alfa Aesar, Ward Hill, MA, USA), water for analysis (Carlo Erba, Cornaredo, Milan, Italy), methanol HPLC grade (Thermo Fisher Scientific, Pittsburg, PA, USA), sodium acetate trihydrate (Merck, Kenilworth, NJ, USA), acetic acid (PENTA, Radiová, Prague, Czech Republic) and hydrochloric acid (HCl) (PENTA, Radiová, Prague, Czech Republic), EDTA-Na_2_.

### 4.2. Microalgal Species and Cultivation Conditions

*Nannochloropsis oculata*, originally provided by the Laboratory of Zoology (Department of Biology, University of Patras, Patras, Greece), was maintained under low light intensity (~30 μmol m^−2^ s^−1^) provided by warm and cold white fluorescent lamps at a rate of 16 h light and 8 h dark at 20 °C in f/2 medium without silicate or vitamins. Approximately 10 mL of the maintenance cultures was used to inoculate Erlenmeyer flasks of 500 mL capacity with 450 mL four-times concentrated f/2 medium without silicate or vitamins. Initial pH was adjusted to 8 with addition of 1 Ν NaOH before the inoculation. Ambient air was provided at a rate of ~2.8 L L^−1^ min^−1^, which was also the sole means of mixing, while ~100 μmol m^−2^ s^−1^ light was provided continuously by 6000 K white LED light bulbs placed below the cultures. After 15 days, the shake-flask cultures were used to inoculate photobioreactors with an operational volume of 1.5 L at an initial Chlorophyll a concentration of 5–6 mg Chl a L^−1^ in four-times concentrated f/2 medium without silicate or vitamins. The photobioreactors were comprised of a cylindrical glass vessel, 13 cm in diameter, with a heat exchanging coil in the middle, acid/base and air inlets on top and a pH/temperature electrode submerged in the working medium at an angle of ~45°. A gas diffuser submerged in the culture was connected to the air inlet providing an aeration rate of 0.9 L L^−1^ min^−1^. Mixing was also provided with a magnetic stirrer. Light was provided with 1 m long 6000 K led tapes, operating at 0.67 Watts and 83 lumen W^−1^ output, wrapped around the operating portion of the photobioreactors. Temperature and pH for each photobioreactor run were controlled with a Hach controller, whereas light period was controlled with a time switch. pH was controlled with 1 N HCl in experiments with pH 6.5 and 8, and with 1 N NaOH in experiments with pH 9.5.

Cultivation conditions were chosen to examine the effects of temperature, pH and light period with Minitab, using a Fractional Factorial Design with Resolution III and 12 total replicates. Chosen sets of conditions were: (A) 35 °C, pH 6.5, 12:12 (light:dark) Light Period, (B) 27.5 °C, pH 8, 12:12 LP, (C) 20 °C, pH 9.5, 12:12 LP, (D) 20 °C, pH 6.5, 24:0 LP, (E) 27.5 °C, pH 8, 24:0 LP and (F) 35 °C, pH 9.5, 24:0 LP. The ranges of pH and temperature were chosen according to the literature, since *Nannochloropsis* has been reported to grow at pH 6–9.5 or higher [36,39], and temperatures as low as 17 °C and as high as 40 °C [18,31]. Even though extreme pH and temperature were expected to not be optimal for growth, their effects on the biomass composition would be valuable in the development of a two-stage cultivation process, in which biomass is initially grown optimally and subsequently exposed to stress in order to accumulate the desired product. For the effect of LP, which is a much less studied parameter, continuous light and 12:12 were the two levels chosen in order to make comparison with the literature possible, since they are the most widely used in published research on *Nannochloropsis.* The alias structure was as follows: the effect of pH was confounded with the LP-Temperature interaction and the effect of temperature was confounded with the LP-pH interaction, whereas the effects of LP and the pH-Temperature interaction were not confounded with any other effects. Whereas pH and Temperature can dramatically influence the growth rate and the biochemical composition of microalgae, LP is mostly relevant to growth since it influences the amount of light available for photosynthesis. Both LP levels chosen provided adequate energy for growth and thus the LP-pH and LP-Temperature interactions can be assumed to be negligible compared to the main effects and the pH-Temperature interaction. Therefore, the experimental design was considered suitable for investigating the effects of the three parameters. Photobioreactor experiments ran for a total of 6 days, with at least 3 experimental runs per set of conditions, with two replicates per condition remaining after removal of botched runs. The 12 remaining experimental runs were used for the model estimation and statistical analysis. Botched runs were experiments in which the temperature and/or the pH reached values were significantly different than their setpoints, due to malfunctions of the control mechanism (for example inadequate cooling or heating, not properly operating acid-base pumps). Optical density at 750 nm, Total Suspended Solids (TSS), nitrate concentration, total phosphorus, concentration, Chlorophyll a concentration and total carotenoids and xanthophylls concentration were monitored daily as described below.

### 4.3. Analytical Measurements

TSS were measured according to *Standard Methods for the Examination of Water and Wastewater* [40], by using 0.5 M ammonium bicarbonate for biomass washing [41] and GF/F grade filters. OD_750_ was measured with a Cary50 UV/VIS, Varian spectrophotometer and used to derive the maximum specific growth rate (μ_max_) during the logarithmic growth phase. Nitrate and total phosphorus concentration was measured daily at the filtered growth medium obtained from the TSS measurement. Nitrates were measured spectroscopically at 220 and 275 nm [40], whereas total phosphorus was measured as orthophosphates with the ascorbic acid method after hydrolyzation under low pH [40].

### 4.4. Biomass Composition Analysis

#### 4.4.1. Biomass Harvesting

At the end of each run, wet biomass was obtained via centrifugation at 3780× *g* for 7 min (Z 366, Hermle AG, Gosheim, Germany), washed with 0.5 M ammonium bicarbonate [41], freeze-dried (Telstar, LyoQuest, Barcelona, Spain) and stored in a desiccator. Ethanol (EtOH)-extracted biomass was also freeze-dried and stored in a desiccator. Both, biomass directly obtained from the experiments and EtOH-extracted biomass were subjected to characterization, as described below.

#### 4.4.2. Moisture and Ash Content Determination

The moisture content of the freeze-dried biomass was determined after drying at 105 °C [40], whereas its ash content was determined after further incineration at 550 °C for 45 min [40].

#### 4.4.3. Protein Content Determination

Protein content was determined with the Semi-micro Kjeldahl method [40]. Specifically, 20 mg of freeze-dried biomass were digested with 7 g K_2_SO_4_, 350 mg H_g_O, 50 mL 3D H_2_O and 10 mL concentrated H_2_SO_4_ at 200 °C for 1 h and at 370 °C for 2 h using a VELP Scientifica Automatic Digestion Unit. Produced ammonium was then converted to free ammonia and captured in 25 mL of 2% boric acid with pH indicators using a VELP Scientifica Kjeldahl Distillation unit. The captured ammonia was quantified with titration using 0.02 N H_2_SO_4_ and converted to protein content with a conversion factor 6.25 [40]. 

#### 4.4.4. Carbohydrate Content Determination

Carbohydrate content was determined with the phenol-sulfuric acid method [42]. Specifically, 5–10 mg of freeze-dried biomass was suspended in 10–100 mL 3D H_2_O. While stirring with a magnet, 1 mL of the suspension was transferred to a glass vial and digested with 1 mL 5% phenol and 5 mL concentrated H_2_SO_4_. Absorption was measured at 490 nm and converted to glucose equivalents with a standard curve [27]. 

#### 4.4.5. Lipid Profiling and Quantification

Lipid content and the lipid profile were determined with one-step in-situ transesterification [43] and subsequent analysis of the derived Fatty acid methyl esters (FAMEs) was made on a GC (7890A, Agilent Technologies Inc., Santa Clara, CA, USA), equipped with a flame ionization detector (FID) and a capillary column (DB–WAX, 10 m × 0.1 mm × 0.1 μm) as previously described [44]. To quantify the produced FAMEs, a reference standard (FAMQ-005, Accustandard, New Haven, CT, USA) and an internal standard solution (C17: 0, Sigma-Aldrich, St. Louis, MO, USA) were used [44].

#### 4.4.6. Pigment Content Determination 

Chlorophyll a and b (Chl a, Chl b) as well as total carotenoids and xanthophylls (C_c+x_) were determined via extraction with *N*,*N*’-dimethylformamide (DMF), at room temperature, for at least 20 min and subsequent spectroscopic estimation according to previous studies [45,46]. For estimation of total pigments in running experiments, 1–3 mL of culture medium was centrifuged at 3780× *g* for 5 min (Z 366, Hermle AG, Gosheim, Germany). Then, the supernatant was discarded, and pigments were extracted with 4 mL DMF. After a second centrifugation, spectroscopic estimation followed. For estimation of pigment content in freeze-dried biomass, 2–3 mg of biomass was extracted with 10 mL DMF. For estimation of pigment contend in ethanolic extracts, 100–300 μL of extract was diluted in a much larger volume of DMF.

### 4.5. Ultrasound Assisted Extraction with 100% EtOH

As described before [3], approximately 20 mg of freeze-dried biomass was extracted with 2 mL 100% EtOH for 15 min in an ultrasonic bath at temperature maintained below 40 °C. Pigment content and reactivity of the extract to the Folin–Ciocalteu assay were measured directly after the extraction, whereas remaining extracts were stored at −18 °C under a nitrogen atmosphere.

### 4.6. Folin–Ciocalteu (F–C) Assay

Reactivity of the ethanolic extracts towards the F–C Assay was measured as described before [4]. More specifically, 100 μL of extract was added to 3D H_2_O to a final volume of 7 mL. Subsequently, 0.5 mL of Folin–Ciocalteu reagent was added. After 1–2 min, 1.5 mL 1.89 M Na_2_CO_3_ solution was added. The volume was finally adjusted to 10 mL with 3D H_2_O. After an incubation of 2 h in the dark, absorption at 760 nm was measured and converted to mg Gallic acid equivalents (GAE) L^−1^ with a standard curve. The results were also expressed as mg GAE per g of biomass DW (mg GAE g^−1^ DW).

### 4.7. Statistical Analysis

One-way ANOVA followed by the Tukey–Kramer Test in Matlab were used to find significant differences between the six experimental conditions, treating each condition as a separate group. Linear regression including center points was performed in Minitab to evaluate the cultivation condition effects on growth parameters and biomass composition. Pearson correlation was used to identify relationships among the extraction parameters and the biomass composition.

## 5. Conclusions

The goal of this study was the investigation of the effects of growth conditions on the growth and biomass composition of *N. oculata*, as well as the evaluation of ethanolic extracts. Three growth parameters, pH, temperature, and light period were evaluated for their effects on growth, biomass composition and fatty acid profile of *N. oculata*. A fractional factorial design was used, with three levels for pH and temperature and two levels for light period. Using the obtained regression models, optimum conditions for growth were estimated to be LP 24:0, pH 9.5 and a temperature of 20 °C; for carbohydrate, protein, FA, and EPA contents LP 12:12, pH 6.5 and a temperature of 20 °C; for Chla content, LP 24:0, pH 8 and a temperature of 27.5 °C; and for C_c+x_ content, LP 24:0, pH 6.5 and a temperature of 35 °C. Although the results provide insight into the effects of growth conditions and help to evaluate previously reported data, precise estimates of the optimal conditions can be obtained with the use of a response surface design. 

Ethanolic extracts were prepared from the obtained biomass, and their reactivity towards the Folin–Ciocalteu assay as well as their pigment content were evaluated. A possible effect of the biomass composition on the level of residual moisture after freeze drying, and, additionally, an effect of residual moisture on the extraction efficiency were implied by Pearson correlation. Finally, in agreement with our previous work, we show a strong correlation between pigment content, especially Chla, with reactivity towards the Folin–Ciocalteu assay.

Overall, the results suggest that further optimization should focus on the effect of light period at moderate pH and low temperatures as well as salinity. Additionally, effects of moisture content on the extraction can be adjusted and studied independently from the biomass composition.

## Figures and Tables

**Table 1:** Specific maximum growth rate, final biomass concentration (AFDW), pigment concentration and nutrient consumption ± the standard deviation.

Conditions *	(A) LP 12:12/ pH 6.5/ T 35 °C	(B) LP 12:12/ pH 8/ T 27.5 °C	(C) LP 12:12/ pH 9.5/ T 20 °C	(D) LP 24:0/ pH 6.5/ T 20 °C	(E) LP 24:0/ pH 8/ T 27.5 °C	(F) LP 24:0/ pH 9.5/ T 35 °C
μ_max_ (day^−1^)	0.00 ± 0 ^b^	0.09 ± 0.03 ^ab^	0.17 ± 0.03 ^ab^	0.18 ± 0.03 ^ab^	0.23 ± 0.06 ^a^	0.07 ± 0.02 ^ab^
TSS (g L^−1^ AFDW)	0.16 ± 0.03 ^c^	0.33 ± 0.01 ^bc^	0.47 ± 0.02 ^ab^	0.42 ± 0.09 ^ab^	0.66 ± 0.02 ^a^	0.35 ± 0.03 ^bc^
Chla (mg L^−1^)	5.17 ± 0.3 ^cd^	7.9 ± 0.59 ^bcd^	9.1 ± 0.05 ^bc^	11.37 ± 6.08 ^b^	19.13 ± 0.47 ^a^	4.21 ± 1.2 ^d^
C_c+x_ (mg L^−1^)	1.95 ± 0.13 ^bc^	2.25 ± 0.21 ^bc^	2.63 ± 0.09 ^bc^	3.27 ± 1.44 ^b^	5.29 ± 0.16 ^a^	1.7 ± 0.32 ^c^
N consumption (mg N L^−1^)	0 ± 0 ^b^	14.89 ± 10.06 ^a^	15.22 ± 5.61 ^a^	18.94 ± 0.43 ^a^	23.60 ± 1.53 ^a^	8.58 ± 9.42 ^a^
P consumption (μg P L^−1^)	4043.35 ± 128.49	4130.57 ± 364.72	4936.64 ± 73.87	4527.11 ± 178.28	4694.49 ± 22.56	4980.89 ± 3.01

LP, Light Period. T, Temperature. TSS, Total Suspended Solids. Chla, Chlorophyll a. C_c+x_, Total Carotenoids and Xanthophylls. N, Nitrogen. P, Phosphorus. * Absence of common superscripts in the same row indicates significant differences (Tukey-Kramer Test).

**Table 2:** Proximate composition AFDW, EPA content and pigment content of the final biomass under the different growth conditions ± the standard deviation.

Conditions *	(A) LP 12:12/ pH 6.5/ T 35 °C	(B) LP 12:12/ pH 8/ T 27.5 °C	(C) LP 12:12/ pH 9.5/ T 20 °C	(D) LP 24:0/ pH 6.5/ T 20 °C	(E) LP 24:0/ pH 8/ T 27.5 °C	(F) LP 24:0/ pH 9.5/ T 35 °C
Carbs % AFDW	8.79 ± 0.2 ^d^	18.02 ± 0.98 ^a^	9.49 ± 0.05 ^cd^	14.2 ± 0.54 ^b^	12.72 ± 1.11 ^bc^	6.73 ± 0.12 ^d^
Protein % AFDW	51.58 ± 1.12 ^a^	56.73 ± 1.85 ^a^	25.49 ± 3.52 ^b^	57.19 ± 4.98 ^a^	47.68 ± 4.72 ^a^	25.58 ± 4.63 ^b^
FA % AFDW	15.21 ± 0.13 ^a^	16.75 ± 0.6 ^a^	8.62 ± 2.3^b^	16.94 ± 0.77 ^a^	14.43 ± 0.11 ^a^	11.92 ± 0.27 ^ab^
Other % **	16.54 ± 1.22 ^a^	1.77 ± 0.9 ^b^	17.38 ± 0.93 ^a^	7.1 ± 4 ^ab^	15.77 ± 4.01 ^a^	19.31 ± 0.64 ^a^
EPA % AFDW	3.36 ± 0.03 ^cd^	4.81 ± 0.02 ^a^	2.36 ± 0.09 ^d^	4.43 ± 0.39 ^ab^	3.5 ± 0.06 ^bc^	1.7 ± 0.21 ^d^
Chla % AFDW	3.42 ± 0.41 ^b^	5.51 ± 0.41 ^a^	2.23 ± 0.2 ^bc^	3.26 ± 0.36 ^b^	6.56 ± 0.03 ^a^	1.26 ± 0.21 ^c^
C_c+x_ % AFDW	1.24 ± 0.13 ^ab^	1.05 ± 0.07 ^abc^	0.45 ± 0.07 ^c^	0.74 ± 0.18 ^bc^	1.58 ± 0.16 ^a^	0.45 ± 0.07 ^c^

LP, Light Period. T, Temperature. Carbs, Carbohydrates. FA, Fatty Acids. EPA, Eicosapentaenoic Acid. Chla, Chlorophyll a. C_c+x_, Total Carotenoids and Xanthophylls. * Absence of common superscripts in the same row indicates significant differences (Tukey–Kramer Test). ** Other compounds in total composition including ash.

**Table 3:** Pigments concentration and gallic acid equivalent concentration in the ethanolic extracts, as well as pigment extraction yield ± the standard deviation.

Conditions *	(A) LP 12:12/ pH 6.5/ T 35 °C	(B) LP 12:12/ pH 8/ T 27.5 °C	(C) LP 12:12/ pH 9.5/ T 20 °C	(D) LP 24:0/ pH 6.5/ T 20 °C	(E) LP 24:0/ pH 8/ T 27.5 °C	(F) LP 24:0/ pH 9.5/ T 35 °C
Chla extract (mg L^−1^)	160.29 ± 39.6 ^ab^	354.58 ± 40.55 ^a^	205.06 ± 169.69 ^ab^	156.25 ± 67.48 ^b^	174 ± 80.96 ^b^	69.33 ± 28.08 ^b^
C_c+x_ extract (mg L^−1^)	58.92 ± 16.4 ^ab^	81.92 ± 17.27 ^a^	42.64 ± 26.78 ^ab^	41.76 ± 15.37 ^b^	48.81 ± 22.92 ^b^	34.41 ± 7.91 ^b^
GAE extract (mg L^−1^)	43.51 ± 16.2 ^ab^	87.08 ± 7.8 ^a^	52.57 ± 38.67 ^ab^	41.5 ± 24.82 ^ab^	51.38 ± 31.28 ^ab^	30.75 ± 7.35 ^b^
% Chla extraction	24.77 ± 4.87 ^ab^	36.29 ± 2.36 ^ab^	41.74 ± 16.09 ^a^	26.9 ± 15.43 ^ab^	14.09 ± 6.85 ^b^	28.15 ± 3.5 ^ab^
% C_c+x_ extraction	24.69 ± 3.97 ^bc^	43.95 ± 8.12 ^abc^	50.29 ± 23.84 ^ab^	32.76 ± 20.12 ^bc^	16.47 ± 10.08 ^c^	43.15 ± 9.83 ^a^

LP, Light Period. T, Temperature. Chla, Chlorophyll a. C_c+x_, Total Carotenoids and Xanthophylls. GAE, Gallic acid equivalents. * Absence of common superscripts in the same row indicates significant differences (Tukey–Kramer Test).

**Table 4:** Optimal growth conditions for the growth parameters and biomass components, estimated from the linear regression models.

Optimized Parameters *	LP	pH	T (°C)
μ_max_ (day^−1^)	24:0	9.5	20
TSS (g L^−1^ AFDW)	24:0	9.5	20
Chla (mg L^−1^)	24:0	9.5	20
C_c+x_ (mg L^−1^)	24:0	9.5	20
Carbs % AFDW	12:12	6.5	20
Protein % AFDW	12:12	6.5	20
FA % AFDW	12:12	6.5	20
EPA % AFDW	12:12	6.5	20
Chla % AFDW	24:0	8.0	27.5
C_c+x_ % AFDW	24:0	6.5	35

LP, Light Period. T, Temperature. TSS, Total Suspended Solids. Chla, Chlorophyll a. C_c+x_, Total Carotenoids and Xanthophylls. Carbs, Carbohydrates. FA, Fatty Acid. EPA, Eicosapentaenoic Acid. * Due to the nature of the design, only corner and center points are used in optimization.

## Data Availability

Data will be made available on request.

## Supplementary Materials

The following supporting information can be downloaded at: https://www.mdpi.com/article/10.3390/md20090552/s1, Table S1: N consumption model; Table S2: P consumption model; Table S3: μ_max_ model; Table S4: TSS AFDW model; Table S5: Chla concentration model; Table S6: C_c+x_ concentration model; Figure S1: Chla concentration factorial plots; Figure S2: C_c+x_ concentration factorial plots; Table S7: Carbohydrate content model; Table S8: Protein content model; Table S9: Lipid content model; Table S10: Others content model; Table S11: EPA content model; Table S12: Chla content model; Table S13: C_c+x_ content model.
