# Supplementary material for: Production of Antioxidants and High Value Biomass from Nannochloropsis oculata: Effects of pH, Temperature and Light Period in Batch Photobioreactors
Source: Mar Drugs. 2022 Aug 29;20(9):552. doi: 10.3390/md20090552 (PMC9505407; doi:10.3390/md20090552)
Supplement: Supplementary file 1 [file marinedrugs-20-00552-s001.zip › marinedrugs-1871685-supplementary.pdf]

Table S1. N consumption model

Coded Coefficients

Term	Effect	Coef	SE Coef	T-Value	P-Value	VIF
Constant		10.83	3.00	3.61	0.011	
LP	8.71	4.36	4.24	1.03	0.344	3.00
pH	2.55	1.27	3.00	0.42	0.686	1.00
T	-12.49	-6.25	3.00	-2.08	0.083	1.00
pH*T	-2.44	-1.22	5.20	-0.24	0.822	3.00
Ct Pt		8.41	5.20	1.62	0.157	1.00

Model Summary

S	R-sq	R-sq(adj)	R-sq(pred)
8.48910	60.71%	27.97%	0.00%

Analysis of Variance

Source	DF	Adj SS	Adj MS	F-Value	P-Value
Model	5	668.12	133.623	1.85	0.237
Linear	3	400.93	133.642	1.85	0.238
LP	1	75.91	75.908	1.05	0.344
pH	1	12.99	12.992	0.18	0.686
T	1	312.03	312.025	4.33	0.083
2-Way Interactions	1	3.98	3.983	0.06	0.822
pH*T	1	3.98	3.983	0.06	0.822
Curvature	1	188.61	188.608	2.62	0.157
Error	6	432.39	72.065		
Total	11	1100.50			

Regression Equation in Uncoded Units

N comsumtpion = 3 + 4.36 LP + 3.8 pH + 0.04 T - 0.109 pH*T + 8.41 Ct Pt

Table S2. P consumption model

Coded Coefficients

Term	Effect	Coef	SE Coef	T-Value	P-Value	VIF
Constant		4654.1	88.3	52.68	0.000	
LP	564	282	125	2.26	0.065	3.00
pH	609.3	304.6	88.3	3.45	0.014	1.00
T	-155.5	-77.8	88.3	-0.88	0.413	1.00
pH*T	-364	-182	153	-1.19	0.279	3.00
Ct Pt		-242	153	-1.58	0.165	1.00

Model Summary

S	R-sq	R-sq(adj)	R-sq(pred)
249.860	78.21%	60.05%	12.83%

Analysis of Variance

Source	DF	Adj SS	Adj MS	F-Value	P-Value
Model	5	1344301	268860	4.31	0.052
Linear	3	1108853	369618	5.92	0.032
LP	1	318007	318007	5.09	0.065
pH	1	742479	742479	11.89	0.014
T	1	48366	48366	0.77	0.413
2-Way Interactions	1	88409	88409	1.42	0.279
pH*T	1	88409	88409	1.42	0.279
Curvature	1	155641	155641	2.49	0.165
Error	6	374580	62430		
Total	11	1718881			

Regression Equation in Uncoded Units

P consumption = -246 + 282 LP + 648 pH + 119 T - 16.2 pH*T - 242 Ct Pt

Table S3. µmax model

Coded Coefficients

Term	Effect	Coef	SE Coef	T-Value	P-Value	VIF
Constant		0.1134	0.0158	7.17	0.000	
LP	0.1435	0.0718	0.0224	3.21	0.018	3.00
pH	0.0111	0.0056	0.0158	0.35	0.737	1.00
T	-0.1198	-0.0599	0.0158	-3.79	0.009	1.00
pH*T	-0.1232	-0.0616	0.0274	-2.25	0.065	3.00
Ct Pt		0.0442	0.0274	1.61	0.157	1.00

Model Summary

S	R-sq	R-sq(adj)	R-sq(pred)
0.0599966	92.68%	86.57%	70.70%

Analysis of Variance

Source	DF	Adj SS	Adj MS	F-Value	P-Value
Model	5	0.055574	0.011115	5.55	0.030
Linear	3	0.049533	0.016511	8.25	0.015
LP	1	0.020595	0.020595	10.29	0.018
pH	1	0.000248	0.000248	0.12	0.737
T	1	0.028690	0.028690	14.34	0.009
2-Way Interactions	1	0.010126	0.010126	5.06	0.065
pH*T	1	0.010126	0.010126	5.06	0.065
Curvature	1	0.005219	0.005219	2.61	0.157
Error	6	0.012008	0.002001		
Total	11	0.067582			

Regression Equation in Uncoded Units

µmax (d-1) = -0.902 + 0.0718 LP + 0.1543 pH + 0.0358 T - 0.00548 pH*T + 0.0442 Ct Pt

Table S4. TSS AFDW model

Coded Coefficients

Term	Effect	Coef	SE Coef	T-Value	P-Value	VIF
Constant		0.3513	0.0212	16.56	0.000	
LP	0.3257	0.1629	0.0300	5.43	0.002	3.00
pH	0.1252	0.0626	0.0212	2.95	0.026	1.00
T	-0.1901	-0.0950	0.0212	-4.48	0.004	1.00
pH*T	-0.2569	-0.1285	0.0367	-3.50	0.013	3.00
Ct Pt		0.1425	0.0367	3.88	0.008	1.00

Model Summary

S	R-sq	R-sq(adj)	R-sq(pred)
0.0447362	82.23%	67.43%	28.93%

Analysis of Variance

Source	DF	Adj SS	Adj MS	F-Value	P-Value
Model	5	0.27327	0.054653	15.18	0.002
Linear	3	0.20968	0.069894	19.42	0.002
LP	1	0.10609	0.106093	29.47	0.002
pH	1	0.03134	0.031336	8.71	0.026
T	1	0.07225	0.072253	20.07	0.004
2-Way Interactions	1	0.04401	0.044010	12.23	0.013
pH*T	1	0.04401	0.044010	12.23	0.013
Curvature	1	0.05412	0.054120	15.04	0.008
Error	6	0.02160	0.003600		
Total	11	0.29486			

Regression Equation in Uncoded Units

TSS AFDW = -2.146 + 0.1629 LP + 0.3558 pH + 0.0787 T - 0.01142 pH*T + 0.1425 Ct Pt

Table S5. Chla concentration model

Coded Coefficients

Term	Effect	Coef	SE Coef	T-Value	P-Value	VIF
Constant		7.179	0.377	19.06	0.000	
LP	11.226	5.613	0.533	10.54	0.000	3.00
pH	-1.039	-0.520	0.377	-1.38	0.217	1.00
T	-4.975	-2.488	0.377	-6.60	0.001	1.00
pH*T	-11.140	-5.570	0.652	-8.54	0.000	3.00
Ct Pt		6.338	0.652	9.72	0.000	1.00

Model Summary

S	R-sq	R-sq(adj)	R-sq(pred)
1.06536	97.66%	95.72%	90.66%

Analysis of Variance

Source	DF	Adj SS	Adj MS	F-Value	P-Value
Model	5	284.834	56.967	50.19	0.000
Linear	3	177.683	59.228	52.18	0.000
LP	1	126.017	126.017	111.03	0.000
pH	1	2.159	2.159	1.90	0.217
T	1	49.507	49.507	43.62	0.001
2-Way Interactions	1	82.738	82.738	72.90	0.000
pH*T	1	82.738	82.738	72.90	0.000
Curvature	1	107.136	107.136	94.39	0.000
Error	6	6.810	1.135		
Total	11	291.644			

Regression Equation in Uncoded Units

mg Chla L-1 = -89.9 + 5.613 LP + 13.27 pH + 3.629 T - 0.4951 pH*T + 6.338 Ct Pt

Table S6. C_{c+x} concentration model

Coded Coefficients

Term	Effect	Coef	SE Coef	T-Value	P-Value	VIF
Constant		2.335	0.101	23.17	0.000	
LP	3.033	1.517	0.142	10.64	0.000	3.00
pH	-0.336	-0.168	0.101	-1.67	0.147	1.00
T	-1.024	-0.512	0.101	-5.08	0.002	1.00
pH*T	-2.945	-1.473	0.175	-8.44	0.000	3.00
Ct Pt		1.435	0.175	8.22	0.000	1.00

Model Summary

S	R-sq	R-sq(adj)	R-sq(pred)
0.284983	97.22%	94.90%	88.87%

Analysis of Variance

Source	DF	Adj SS	Adj MS	F-Value	P-Value
Model	5	17.0316	3.40631	41.94	0.000
Linear	3	11.5229	3.84097	47.29	0.000
LP	1	9.1993	9.19929	113.27	0.000
pH	1	0.2257	0.22575	2.78	0.147
T	1	2.0979	2.09787	25.83	0.002
2-Way Interactions	1	5.7826	5.78263	71.20	0.000
pH*T	1	5.7826	5.78263	71.20	0.000
Curvature	1	5.4932	5.49320	67.64	0.000
Error	6	0.4873	0.08122		
Total	11	17.5188			

Regression Equation in Uncoded Units

mg Cc+x L-1 = -23.69 + 1.517 LP + 3.488 pH + 0.979 T - 0.1309 pH*T + 1.435 Ct Pt

Figure S1. Chla concentration factorial plots

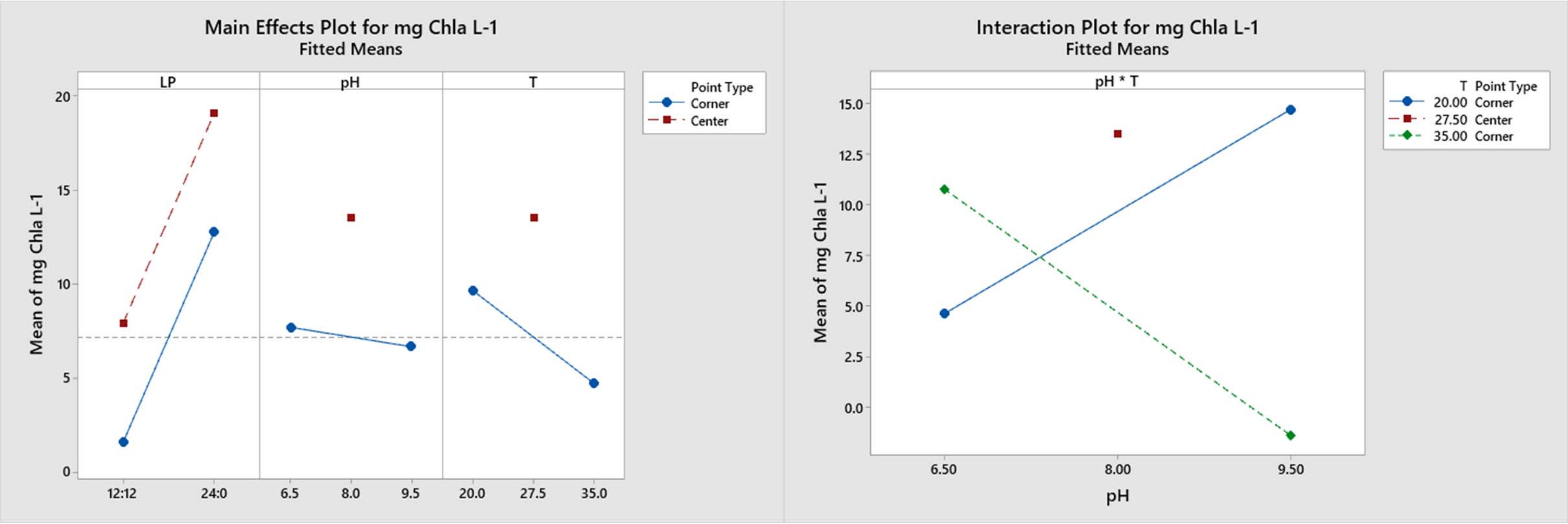

Figure S2. C_{c+x} concentration factorial plots

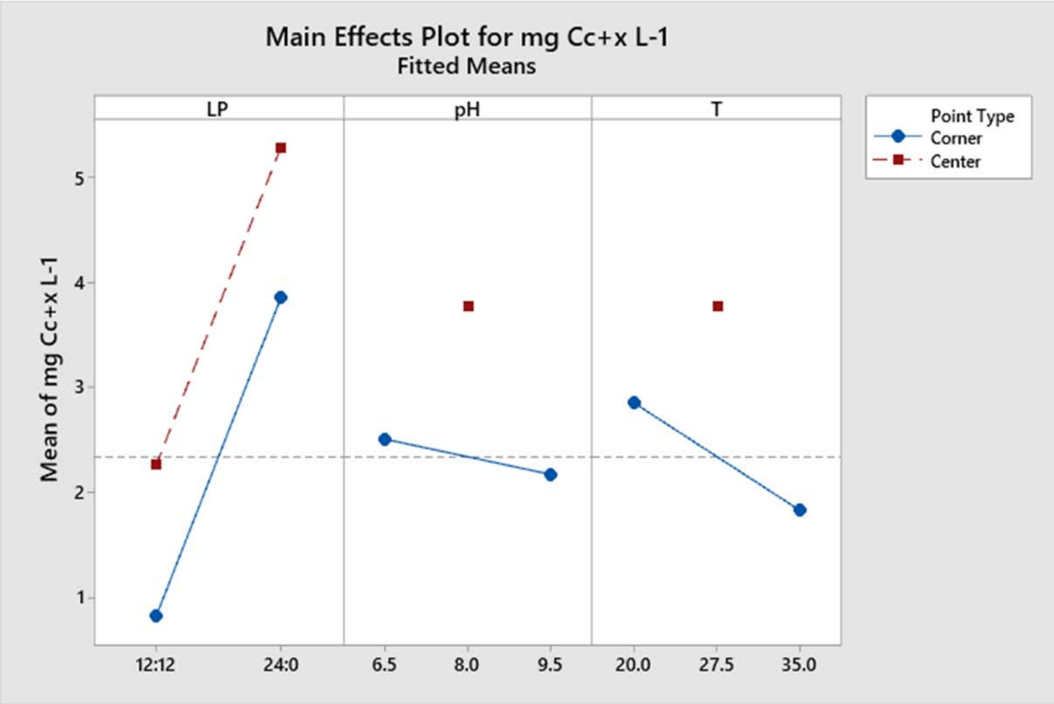

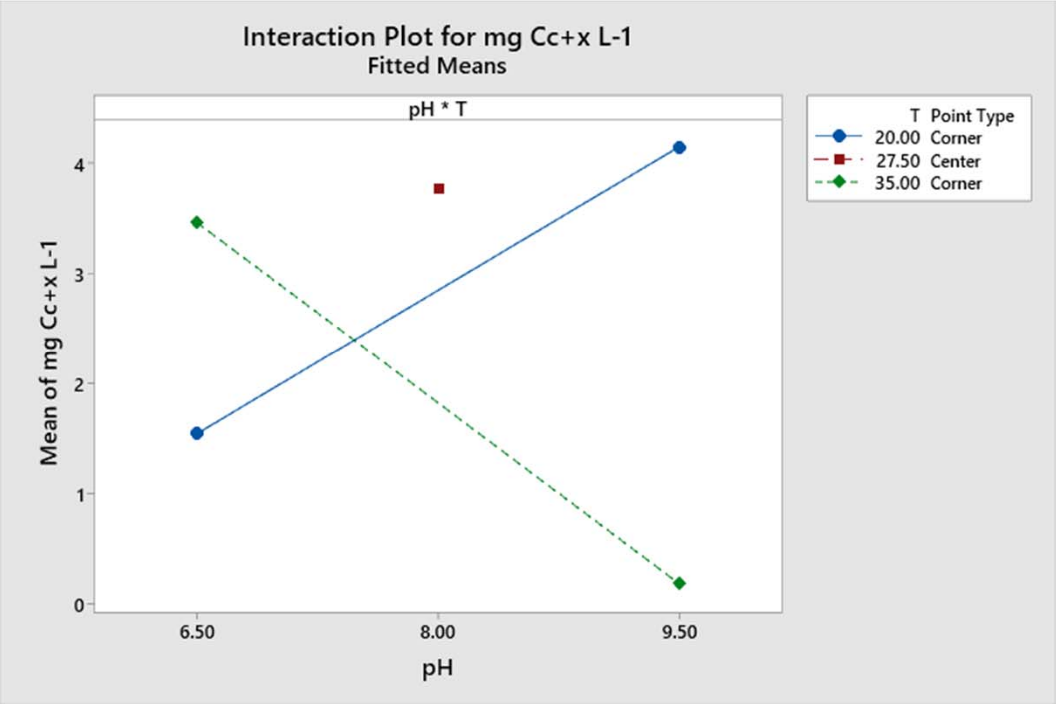

Table S7. Carbohydrate content model

Coded Coefficients

Term	Effect	Coef	SE Coef	T-Value	P-Value	VIF
Constant		9.804	0.326	30.12	0.000	
LP	-5.295	-2.648	0.460	-5.75	0.001	3.00
pH	-3.385	-1.693	0.326	-5.20	0.002	1.00
T	-4.084	-2.042	0.326	-6.27	0.001	1.00
pH*T	6.625	3.312	0.564	5.88	0.001	3.00
Ct Pt		5.566	0.564	9.87	0.000	1.00

Model Summary

S	R-sq	R-sq(adj)	R-sq(pred)
0.920674	97.10%	94.69%	88.41%

Analysis of Variance

Source	DF	Adj SS	Adj MS	F-Value	P-Value
Model	5	170.461	34.0922	40.22	0.000
Linear	3	84.310	28.1034	33.15	0.000
LP	1	28.040	28.0397	33.08	0.001
pH	1	22.918	22.9177	27.04	0.002
T	1	33.353	33.3527	39.35	0.001
2-Way Interactions	1	29.258	29.2583	34.52	0.001
pH*T	1	29.258	29.2583	34.52	0.001
Curvature	1	82.615	82.6155	97.47	0.000
Error	6	5.086	0.8476		
Total	11	175.547			

Regression Equation in Uncoded Units

Carb% AFDW = 91.1 - 2.648 LP - 9.23 pH - 2.628 T + 0.2944 pH*T + 5.566 Ct Pt

Table S8. Protein content model

Coded Coefficients

Term	Effect	Coef	SE Coef	T-Value	P-Value	VIF
Constant		39.96	1.89	21.16	0.000	
LP	-9.05	-4.53	2.67	-1.69	0.141	3.00
pH	-28.85	-14.42	1.89	-7.64	0.000	1.00
T	-2.76	-1.38	1.89	-0.73	0.493	1.00
pH*T	11.90	5.95	3.27	1.82	0.119	3.00
Ct Pt		12.25	3.27	3.74	0.010	1.00

Model Summary

S	R-sq	R-sq(adj)	R-sq(pred)
5.34244	92.71%	86.64%	70.84%

Analysis of Variance

Source	DF	Adj SS	Adj MS	F-Value	P-Value
Model	5	2177.87	435.57	15.26	0.002
Linear	3	1761.81	587.27	20.58	0.001
LP	1	81.98	81.98	2.87	0.141
pH	1	1664.60	1664.60	58.32	0.000
T	1	15.23	15.23	0.53	0.493
2-Way Interactions	1	94.43	94.43	3.31	0.119
pH*T	1	94.43	94.43	3.31	0.119
Curvature	1	399.85	399.85	14.01	0.010
Error	6	171.25	28.54		
Total	11	2349.12			

Regression Equation in Uncoded Units

% Protein AFDW = 238.3 - 4.53 LP - 24.16 pH - 4.42 T + 0.529 pH*T + 12.25 Ct Pt

Table S9. Lipid content model

Coded Coefficients

Term	Effect	Coef	SE Coef	T-Value	P-Value	VIF
Constant		13.174	0.514	25.64	0.000	
LP	-2.317	-1.159	0.727	-1.59	0.162	3.00
pH	-5.803	-2.901	0.514	-5.65	0.001	1.00
T	0.785	0.393	0.514	0.76	0.474	1.00
pH*T	4.830	2.415	0.890	2.71	0.035	3.00
Ct Pt		2.419	0.890	2.72	0.035	1.00

Model Summary

S	R-sq	R-sq(adj)	R-sq(pred)
1.45341	88.96%	79.77%	55.86%

Analysis of Variance

Source	DF	Adj SS	Adj MS	F-Value	P-Value
Model	5	102.177	20.435	9.67	0.008
Linear	3	73.952	24.651	11.67	0.006
LP	1	5.369	5.369	2.54	0.162
pH	1	67.348	67.348	31.88	0.001
T	1	1.234	1.234	0.58	0.474
2-Way Interactions	1	15.550	15.550	7.36	0.035
pH*T	1	15.550	15.550	7.36	0.035
Curvature	1	15.600	15.600	7.39	0.035
Error	6	12.674	2.112		
Total	11	114.851			

Regression Equation in Uncoded Units

% FA AFDW = 74.4 - 1.159 LP - 7.84 pH - 1.665 T + 0.2147 pH*T + 2.419 Ct Pt

Table S10. Others content model

Coded Coefficients

Term	Effect	Coef	SE Coef	T-Value	P-Value	VIF
Constant		15.08	1.22	12.38	0.000	
LP	14.00	7.00	1.72	4.07	0.007	3.00
pH	6.52	3.26	1.22	2.68	0.037	1.00
T	5.68	2.84	1.22	2.33	0.058	1.00
pH*T	-17.76	-8.88	2.11	-4.21	0.006	3.00
Ct Pt		-6.31	2.11	-2.99	0.024	1.00

Model Summary

S	R-sq	R-sq(adj)	R-sq(pred)
3.44442	87.09%	76.33%	48.36%

Analysis of Variance

Source	DF	Adj SS	Adj MS	F-Value	P-Value
Model	5	480.18	96.04	8.09	0.012
Linear	3	345.82	115.27	9.72	0.010
LP	1	196.06	196.06	16.53	0.007
pH	1	85.14	85.14	7.18	0.037
T	1	64.62	64.62	5.45	0.058
2-Way Interactions	1	210.17	210.17	17.72	0.006
pH*T	1	210.17	210.17	17.72	0.006
Curvature	1	106.19	106.19	8.95	0.024
Error	6	71.18	11.86		
Total	11	551.37			

Regression Equation in Uncoded Units

Others% = -186.3 + 7.00 LP + 23.88 pH + 6.69 T - 0.789 pH*T - 6.31 Ct Pt

Table S11. EPA content model

Coded Coefficients

Term	Effect	Coef	SE Coef	T-Value	P-Value	VIF
Constant		2.9611	0.0931	31.81	0.000	
LP	-1.308	-0.654	0.132	-4.97	0.003	3.00
pH	-1.8682	-0.9341	0.0931	-10.03	0.000	1.00
T	-0.8659	-0.4330	0.0931	-4.65	0.004	1.00
pH*T	1.510	0.755	0.161	4.68	0.003	3.00
Ct Pt		1.195	0.161	7.41	0.000	1.00

Model Summary

S	R-sq	R-sq(adj)	R-sq(pred)
0.263312	97.13%	94.74%	88.52%

Analysis of Variance

Source	DF	Adj SS	Adj MS	F-Value	P-Value
Model	5	14.0810	2.81620	40.62	0.000
Linear	3	10.1908	3.39693	48.99	0.000
LP	1	1.7106	1.71060	24.67	0.003
pH	1	6.9805	6.98053	100.68	0.000
T	1	1.4997	1.49965	21.63	0.004
2-Way Interactions	1	1.5202	1.52022	21.93	0.003
pH*T	1	1.5202	1.52022	21.93	0.003
Curvature	1	3.8085	3.80847	54.93	0.000
Error	6	0.4160	0.06933		
Total	11	14.4970			

Regression Equation in Uncoded Units

% EPA AFDW = 24.30 - 0.654 LP - 2.468 pH - 0.595 T + 0.0671 pH*T + 1.195 Ct Pt

Table S12. Chla content model

Coded Coefficients

Term	Effect	Coef	SE Coef	T-Value	P-Value	VIF
Constant		2.543	0.153	16.67	0.000	
LP	1.053	0.527	0.216	2.44	0.050	3.00
pH	-1.593	-0.796	0.153	-5.22	0.002	1.00
T	-0.405	-0.203	0.153	-1.33	0.232	1.00
pH*T	-1.621	-0.811	0.264	-3.07	0.022	3.00
Ct Pt		3.495	0.264	13.23	0.000	1.00

Model Summary

S	R-sq	R-sq(adj)	R-sq(pred)
0.431412	97.27%	94.99%	89.06%

Analysis of Variance

Source	DF	Adj SS	Adj MS	F-Value	P-Value
Model	5	39.7251	7.9450	42.69	0.000
Linear	3	6.5114	2.1705	11.66	0.006
LP	1	1.1092	1.1092	5.96	0.050
pH	1	5.0736	5.0736	27.26	0.002
T	1	0.3286	0.3286	1.77	0.232
2-Way Interactions	1	1.7526	1.7526	9.42	0.022
pH*T	1	1.7526	1.7526	9.42	0.022
Curvature	1	32.5681	32.5681	174.99	0.000
Error	6	1.1167	0.1861		
Total	11	40.8418			

Regression Equation in Uncoded Units

Chla % AFDW = -8.32 + 0.527 LP + 1.451 pH + 0.549 T - 0.0721 pH*T + 3.495 Ct Pt

Table S13. C_{c+x} content model

Coded Coefficients

Term	Effect	Coef	SE Coef	T-Value	P-Value	VIF
Constant		0.7209	0.0614	11.74	0.000	
LP	0.5290	0.2645	0.0868	3.05	0.023	3.00
pH	-0.5444	-0.2722	0.0614	-4.43	0.004	1.00
T	0.2488	0.1244	0.0614	2.03	0.089	1.00
pH*T	-0.784	-0.392	0.106	-3.68	0.010	3.00
Ct Pt		0.593	0.106	5.57	0.001	1.00

Model Summary

S	R-sq	R-sq(adj)	R-sq(pred)
0.173700	91.93%	85.21%	67.73%

Analysis of Variance

Source	DF	Adj SS	Adj MS	F-Value	P-Value
Model	5	2.0631	0.41262	13.68	0.003
Linear	3	0.9965	0.33217	11.01	0.007
LP	1	0.2798	0.27985	9.28	0.023
pH	1	0.5928	0.59284	19.65	0.004
T	1	0.1238	0.12381	4.10	0.089
2-Way Interactions	1	0.4093	0.40934	13.57	0.010
pH*T	1	0.4093	0.40934	13.57	0.010
Curvature	1	0.9370	0.93700	31.06	0.001
Error	6	0.1810	0.03017		
Total	11	2.2441			

Regression Equation in Uncoded Units

Car % AFDW = -5.95 + 0.2645 LP + 0.776 pH + 0.2952 T - 0.03483 pH*T + 0.593 Ct Pt
